# Peripheral Solitary Osteoma of the Zygomatic Arch: A Case Report and Literature Review

**DOI:** 10.2174/1874210601711010120

**Published:** 2017-02-28

**Authors:** Thomas Starch-Jensen

**Affiliations:** Department of Oral and Maxillofacial Surgery, Aalborg University Hospital, Aalborg, Denmark

**Keywords:** Dentistry, Diagnostic imaging, Facial bones, General surgery, Neoplasms, Osteoma

## Abstract

Osteoma is a benign slow-growing osteogenic neoplasm commonly occurring in the craniofacial skeleton, characterized by the proliferation of compact and/or cancellous bone. Osteomas may be peripheral, central, or extraskeletal. Peripheral osteomas arise from the periosteum and are quite uncommon in the jaw bones. The exact aetiology and pathogenesis of peripheral osteoma are unknown. Clinically, peripheral osteomas are usually asymptomatic, but depending on the location and size of the lesion, it may cause swelling, pain, esthetic disfigurement and functional impairment. On radiological imaging, a peripheral osteoma appears often as well-circumscribed, round to oval, pedunculated radiopaque mass attached to the cortex by a broad base or a pedicle. Asymptomatic osteomas are treated conservatively, while surgical excision is indicated when the lesion is symptomatic, actively growing, or for cosmetic reasons. Histologically, osteomas are composed of a normal-appearing, dense mass of lamellar bone. Recurrence of peripheral osteoma after surgical removal is extremely rare and there are no reports of malignant transformation. A review of the literature disclosed only 7 well-documented cases of peripheral osteoma located at the zygomatic bone. The purpose of this article is to present the clinical, radiographic, surgical and histological features of a solitary peripheral osteoma of the left zygomatic arch in a 55-year-old woman and to review the literature about this uncommon pathologic entity.

## INTRODUCTION

Osteoma is a benign slow growing neoplasm characterized by the proliferation of compact or cancellous bone, and one of the most common tumors of the nose and paranasal sinuses [1]. It was first recognized as a tumor by Jaffe in 1935 [2]. Multiple osteomas are mainly associated with Gardner's syndrome while solitary osteomas can be further classified based on the location as: (1) the central osteoma arising from the endosteum; (2) the peripheral osteoma deriving from the periosteum and (3) the extraskeletal soft tissue osteoma, which usually develops within the muscles [2]. The etiology of the osteoma has not been established, but several explanations have been suggested for the origin of these neoplasms such as osteogenic, traumatic, and infective sources [1]. Females present a higher incidence rate without predilection for any specific age range [3]. The majority of osteomas are peripheral, while central osteomas in the craniofacial skeleton are uncommon [4]. Peripheral osteomas in general present as a solitary, slowly and asymptomatic continuous growing hard mass protruding from the periphery of the surface of bone [5]. Osteoma in the facial skeleton is often detected incidentally on routine radiographic examination or when it causes facial asymmetry, pain, limitation or deviation of the mandible on opening, or dysfunction [6-8]. Radiographically, peripheral osteomas usually present as a round or oval well-circumscribed radiopaque bone-thickening lesion whose base is united with the underlying cortical bone, often as a pedunculated lesion but may also have a broad-base [7]. The slow growth of an osteomas justified a conservative approach toward an asymptomatic lesion, whereas surgical removal of osteomas is indicated when the lesion is symptomatic, actively growing, or causing esthetic disfigurement and functional impairment. Histopathologically, the compact osteomas comprises of dense, compact bone with a few marrow spaces and with only a few osteons [8]. Osteons are the structural unit of compact bone consisting of concentric layers, or lamellae, of compact bone tissue that surround the haversian canal. The cancellous osteoma is characterized by bony trabeculae and a fibro fatty marrow enclosing osteoblast and with architecture resembling mature bone [8]. Osteoma is a benign tumor with low tendency to recur when treated by an adequate surgical technique [8]. Although surgical excision is recommended for growing symptomatic osteomas, there are no reports of malignant transformations in reviewed literature in English language [8].

The purpose of presenting this case report is to increase the number of reported cases of zygomatic osteomas and to summarize the current knowledge of this uncommon maxillofacial lesion.

## CASE PRESENTATION

A 55-year-old woman was referred to the Department of Oral and Maxillofacial Surgery, Aalborg University Hospital, Denmark, due to a long-lasting increasing dull pain from the left side of her face. There was no previously history of facial trauma and her medical history did not contain any known pathology of the intestines.

**Fig. (1):**
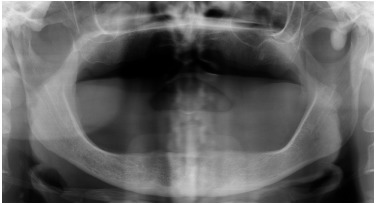
Panoramic radiographic visualizing a pedunculated osseous lesion originating from the left zygomatic arch.

**Fig. (2):**
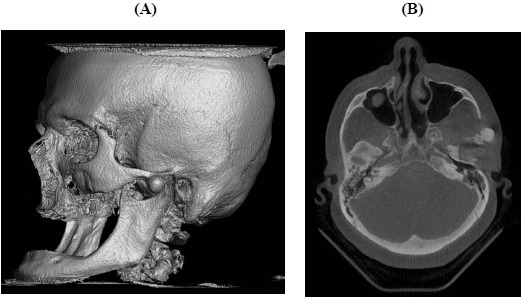
Cone Beam Computed Tomography scan. (**A**) Axial image showing a round well-circumscribed radiopaque lesion originating from the lateral surface of the left zygomatic arch. (**B**) Three-dimensional reconstruction image visualizing a well-circumscribed radiopaque structure located on the lateral border of the left zygomatic arch.

Extraorally, a discrete swelling was observed anterior to the left temporomandibular joint. There were no signs of inflammation. The temporalis and masseter muscles were tender on palpation, with normal range of motion. Intraorally, the patient was edentulous with well-functioning removable prosthesis. A panoramic radiographic exam showed the presence of a pedicled osseous lesion originating from the left zygomatic arch (Fig. **1**). A cone beam computed tomography confirmed the presence of a 1.2 X 1.4 cm well-circumscribed radiopaque structure located on the lateral border of the left zygomatic arch (Fig. **2**). On the basis of the clinical and radiographic findings, a working diagnosis of peripheral osteoma of the zygoma arch was made. Due to increasing pain and cosmetic reasons, the decision was made to surgically remove the tumor.

**Fig. (3):**
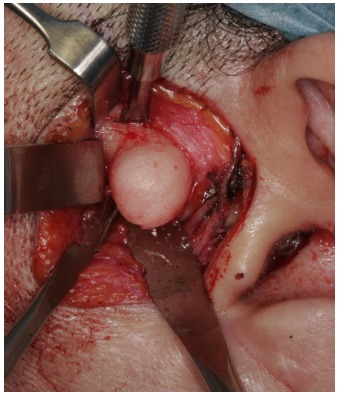
Intraoperative clinical photo of the exposed peripheral osteoma on the zygomatic arch.

**Fig. (4):**
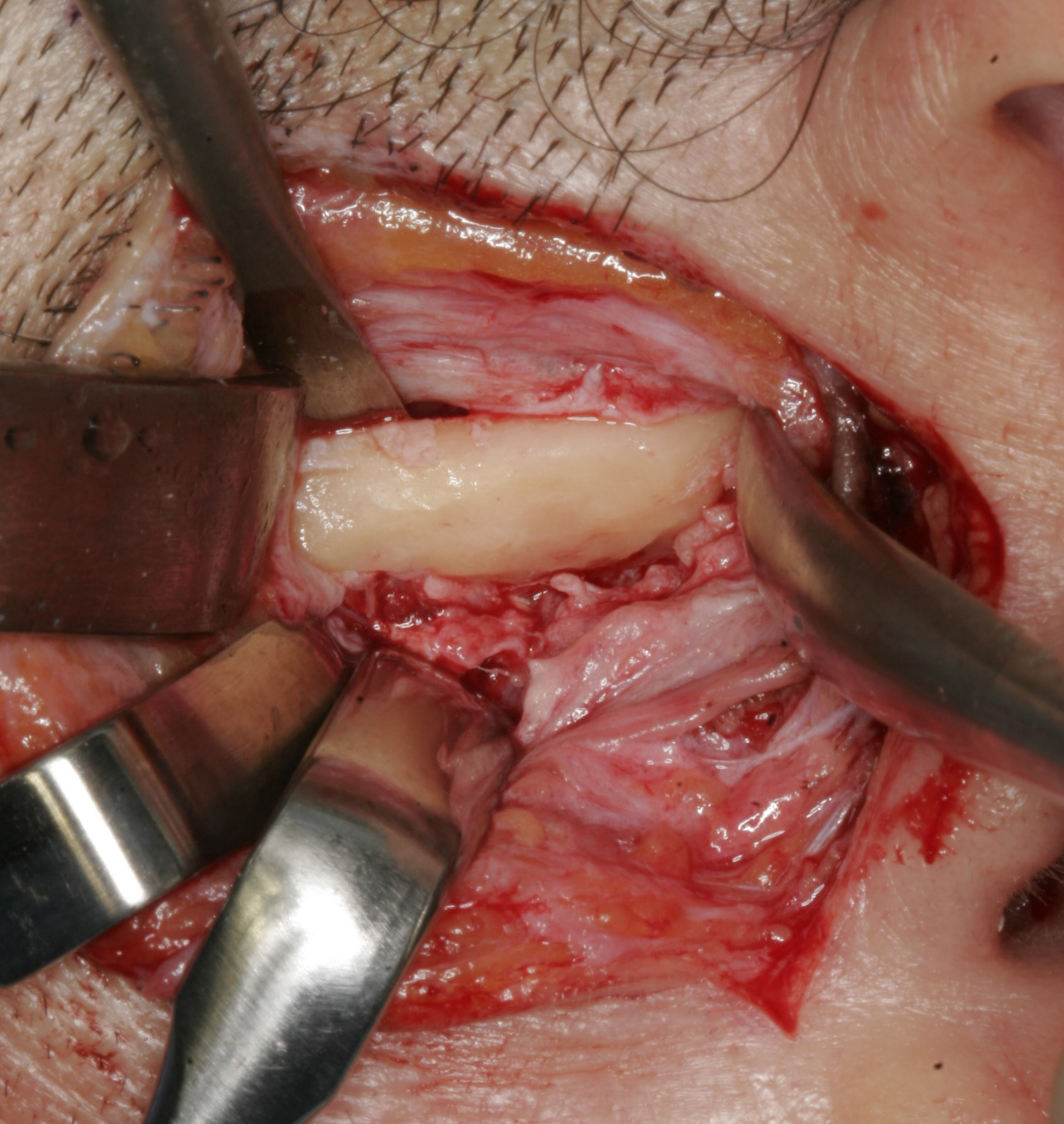
Intraoperative clinical photo after resection of the peripheral osteoma and recontouring of the zygomatic arch.

In general anaesthesia, a preauricular incision through the skin and subcutaneous connective tissue was performed. The tissue was reflected by blunt dissection above the zygomatic arch to the level of the superficial layer of the temporalis fascia. The superficial temporal vessels were retracted anteriorly with the skin flap and nerve stimulator was used during the surgical procedure to detect branches of the facial nerve. After incision of the superficial temporal fascia over the zygomatic arch and dissection of the periosteum from the lateral portion of the zygomatic arch, a complete view of the lesion was obtained and the temporal branch of the facial nerve was carefully protected with a retractor within the superficial layer of the temporalis fascia. The pedunculated bony structure was removed with bur and chisel (Fig. **3**). Finally, surgical recontouring of the zygomatic arch was performed (Fig. **4**). Healing was uneventful. Histopathologic examination revealed a well-circumscribed mass composed of dense lamellar bone, compatible with osteoma.

**Fig. (5):**
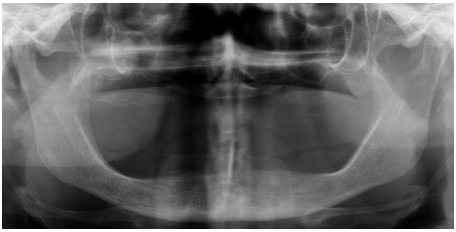
Panoramic radiographic after one year disclosing no recurrence of the peripheral osteoma on left the zygomatic arch.

There was no clinical or radiographic evidence of recurrence on the one year follow-up examination (Fig. **5**). The patient had normal neurovascular function, but minor pain from her temporomandibular joint and muscles persisted.

## DISCUSSION

We present a 55 years old woman with a peripheral zygomatic osteoma and summarize the current knowledge about this uncommon benign osteogenic neoplasm. Peripheral osteomas are common in the craniofacial region, while osteoma of the zygoma is extremely uncommon. A search of the literature identified only 7 well-documented cases of peripheral osteomas of the zygoma (Table **1**) [9-15]. The majority of these cases display as a well-defined circular pedunculated radiopaque osseous lesion origination from either the zygomatic bone or arch. Peripheral osteoma in the facial skeleton is often detected incidentally on routine radiographic examination due to an asymptomatic slow growth rate. However, only one of the previous cases was found incidentally in relation to a radiological examination prior to installation of dental implants [12]. All the other cases was examined due to either pain, swelling, facial asymmetry, cosmetic disfigurement or functional impairment, but in some of the reported cases, the peripheral osteoma has debuted several years earlier and recently increased in size [11, 14]. Hence, the actual symptoms should not be related to the growth of the peripheral zygomatic osteoma, but more likely to the compression from the lesion to the adjacent anatomical structures, which is in accordance with the symptoms of our case.

Usually, a conventional radiograph is sufficient to make a diagnosis of a peripheral osteoma, but computed tomography with three-dimensional reconstruction is the best imaging modality for precise localization of the osteoma and treatment planning prior to surgical removal. An individualized approach for the management of osteomas is recommended considering the size and location of the tumor. Smaller asymptomatic peripheral osteomas generally do not require any treatment, whereas surgical intervention is indicated when the lesion is larger, symptomatic, actively growing or causes functional impairment. Surgical removal of a peripheral zygomatic osteoma by an intraoral approach is desirable since it avoids facial scarring but the ease of accessibility is limited. Therefore, an extraoral approach is frequently used. However, the decision regarding the type of surgical approach should depend upon the anatomical location of the osteoma, risk of complications and cosmetic patient demands. Consequently, the surgical approach to remove a peripheral osteoma of the zygoma should be case specific and the intraoral approach is preferable when possible, mainly for cosmetic reasons [16]. Surgical treatment of a symptomatic osteoma consists of complete removal at the base where it unites with the cortical bone and recurrence of peripheral osteomas of the zygoma after surgical excision is rare, with a single case occurred 10 years after surgical treatment [15]. There are no reports of malignant transformation of peripheral osteoma in reviewed literature in English language, but regular clinical and radiographic follow-up examination is advised.

Clinically, peripheral osteoma should be differentiated from several pathologies, such as exostoses, osteoblastoma, osteoid osteoma, late-stage central ossifying fibroma or complex odontoma [17].

The presence of craniofacial osteomas may be a sign of the presence of Gardner's syndrome which is an autosomal dominant syndrome characterized by the presence of polyps in the gastrointestinal area, several osteomas of the skull and face, soft tissue tumors, skin tumors, and supernumerary multiple impacted teeth. Since the osteomas develop before the colorectal polyposis, early recognition of the syndrome is very important to the prognosis of the disease [16]. Therefore, General Dental Practitioners should be aware of Gardner´s syndrome if they diagnose a patient with impacted teeth and osteomas of the jaws.

In this case, surgical resection performed was completely effective and no complications with the pre-auricular approach have been observed after a follow-up of one year.

## CONCLUSION

A case of a solitary peripheral zygomatic osteoma has been presented and the current knowledge about this uncommon entity has been discussed. The clinical and radiographic appearance of a solitary peripheral osteoma of the zygoma is very characteristic. The present case was not part of Gardner syndrome since no pathology of the intestines or impacted teeth was discovered. The association of osteoma with Gardner's syndrome must always be kept in mind and General Dental Practitioners should have knowledge of this serious disease.

## Figures and Tables

**Table 1:** Clinical features of 7 previous published cases of zygoma osteoma.

**Case**	**Age/** **Gender**	**Clinical symptoms**	**Radiographic findings**	**Treatment**	**Complications**	**Histopathological examination**	**Follow-up**	**Relapse**	**References**
1	25/♂	Restricted mandibular opening	Pedunculated osseous lesion medial to the zygomatic arch	Surgical resection by intraoral approach	Healing was uneventful	Normal bone with medullary fibrosis	8 weeks	No	Boland *et al*., 1983 [9]
2	55/♀	Left ear was closing	Pedunculated osseous lesion lateral to the zygomatic arch	Surgical resection by extraoral approach	Healing was uneventful	Normal cortical bone	ND	ND	Furlaneto *et al*., 2004 [10]
3	20/♂	Itchy facial swelling	Pedunculated osseous radiopaque mass on the zygoma	Surgical resection by extraoral approach	Healing was uneventful	Dense compact bone with sparse marrow	6 months	No	Akinmoladun *et al*., 2007 [11]
4	61/♀	None	Pedunculated osseous lesion on the zygomatic arch	Observation	ND	Not performed	ND	ND	Durao *et al*., 2012 [12]
5	62/♂	Pain and tenderness	A well-defined circular radiolucent lesion at the zygoma	Surgical resection by extraoral approach	ND	Well-vascularized stroma and immature bone	ND	ND	Mintz *et al*., 2013 [13]
6	32/♀	Painful swelling	Firm bony swelling above the zygomatic arch	Surgical resection by extraoral approach	Healing was uneventful	Cancellous-type osteoma	7 years	No	Quintans *et al*., 2013 [14]
7	41/♂	Painless firm mass	Lobulated bony mass protruding from outer cortex of the zygoma	Surgical resection by intraoral approach	Healing was uneventful	Normal mature compact bone	1 month	No	Kim *et al*. 2015 [15]

Abbreviations: ND: no data.
